# Epithelium-preserving stricturotomy is effective for improving postoperative benign anastomotic stricture associated with colorectal surgery

**DOI:** 10.1055/a-2592-3133

**Published:** 2025-05-16

**Authors:** Xiaoling Hong, Dezheng Lin, Dejun Fan, Xutao Lin, Junguo Chen, Jiancong Hu

**Affiliations:** 1 373651 Department of General Surgery (Endoscopic Center), Sun Yat-sen University Sixth Affiliated Hospital, Guangzhou, China; 2 373651 Department of Endoscopic Surgery, Sun Yat-sen University Sixth Affiliated Hospital, Guangzhou, China; 3 373651 Department of Thoracic Surgery, Sun Yat-sen University Sixth Affiliated Hospital, Guangzhou, China

**Keywords:** Endoscopy Lower GI Tract, Colorectal cancer, Endoscopy Upper GI Tract, Benign strictures

## Abstract

**Background and study aims:**

We analyzed a large sample of patients with colorectal cancer (CRC) treated with endoscopic stricturotomy (ESt) for postoperative benign anastomotic stricture (POBAS) and explored risk factors for stricture recurrence (restricture). We aimed to provide data on the long-term outcomes of ESt and support for optimizing ESt in treating and preventing POBAS recurrence.

**Patients and methods:**

This retrospective study included 152 consecutive patients with CRC diagnosed with POBAS and treated by ESt at our center from April 2013 to April 2023. The primary outcome was stricture recurrence. Secondary outcomes were the technical success rate, postoperative adverse events (AEs), and restricture-free survival (RFS). Risk factors for stricture recurrence were explored.

**Results:**

Of the 152 patients, 94.1% (143/152) achieved technical success after the first ESt. Twenty-two patients (17.5%) were diagnosed with recurrent stricture among 126 initial successful patients with follow-up. Anastomotic stricture length ≥ 1 cm and non-preservation of intestinal epithelium during ESt were independent risk factors for recurrence ( *P* < 0.05). The cumulative RFS rate was 82.53%.

**Conclusions:**

Anastomotic stricture length ≥ 1 cm and non-epithelium preservatoin at ESt were independent risk factors for restricture after ESt in POBAS patients. These two factors may help predict risk of POBAS recurrence and provide reliable evidence for developing personalized treatment plans for patients.

## Introduction

The most recent cancer statistics report that colorectal cancer (CRC) is the third most common malignant tumor and the second leading cause of cancer death 1 . One of the most common complications of rectal cancer surgery is postoperative benign anastomotic stricture (POBAS), which occurs in 8.7% to 13% of patients 2 . Endoscopic stricturotomy (ESt) is an effective novel technology that somewhat improves quality of life for POBAS patients 3 . ESt has surgery-free survival and morbidity comparable to ileocolonic resection. However, some patients experience stricture recurrence (restricture) after ESt 4 5 . Accordingly, there is a need for effective predictors and treatment options to prevent restricture, which is critical for patients with post-CRC anastomotic stricture.

Previous studies have analyzed factors associated with anastomotic stricture occurrence or recurrence from the perspective of anastomotic factors and endoscopic treatment methods. For example, anastomosis methodologies (stapled, hand-sewn, end-to-end, end-to-side, side-to-side), anastomosis location, radiotherapy, anastomotic leakage, and left colic artery preservation may influence incidence of anastomotic stricture following radical CRC surgery 2 6 . Risk of recurrent strictures requiring further procedures or surgery appears to be lower with ESt than with endoscopic balloon dilation (EBD) in gastrointestinal anastomosis. ESt is currently considered a good choice for treating POBAS. However, risk factors for failure of ESt and stricture recurrence remain unknown. Preoperative radiotherapy and stricture length and severity are factors associated with recurrent strictures in EBD. We previously reported that anastomotic stricture length ≥ 1 cm was an independent risk factor for restricture after ESt 4 .

ESt can achieve more complete stricture disruption than EBD, where there is a lower risk recurrence of scar formation because the scar tissue is excised in an arc from the incision along the lumen during ESt 7 . However, a standard ESt procedure has not been established. Some endoscopists remove scar tissue from the anastomosis completely, whereas others make deep longitudinal incisions to preserve epithelial tissue between the incisions. This difference in surgical approach has been explored in hemorrhoid surgery. Huang et al. reported that modified selective hemorrhoidectomy combined with anal epithelial retention could reduce the anal stricture rate 8 . Deep longitudinal incisions to preserve the epithelial tissue in ESt are similar to the anal epithelial retention method in hemorrhoidectomy. Therefore, we speculated about whether preserving the intestinal epithelium during ESt might influence anastomotic stricture recurrence.

In this study, we retrospectively collected clinical data from patients who underwent radical CRC surgery and were admitted to our hospital for ESt treatment due to POBAS from April 2013 to April 2023. We will analyzed long-term outcomes of ESt and risk factors for recurrent stricture in POBAS patients after ESt.

## Patients and methods

### Data sources

This study was approved by the Institutional Review Board of the Sixth Affiliated Hospital, Sun Yat-sen University. In this retrospective study, patients who underwent radical CRC surgery and were admitted to our hospital for ESt treatment due to POBAS between April 2013 and April 2023 were identified. Anastomotic stricture was defined as inability of a direct 12-mm endoscope to pass through the anastomosis with or without intestinal obstruction.

Inclusion criteria were: 1) age ≥ 18 years; 2) CRC confirmed by pathology before treatment; 3) receipt of surgical treatment for CRC resection; 4) colonoscopy performed to diagnose POBAS; and 5) stricture initially treated with ESt. Exclusion criteria were: 1) abnormal coagulation function; and 2) severe anastomotic leakage (such as active perianastomotic abscess).

### Demographic and clinical data

Information about the following patient demographic and clinical characteristics was retrieved: age, sex, body mass index (BMI), neoadjuvant therapy (neoadjuvant chemotherapy, neoadjuvant radiotherapy, or neoadjuvant chemoradiotherapy), anastomosis location (distance to the anal verge), postoperative anastomotic leakage, median time from radical CRC surgery to first anastomotic stricture (months), anastomotic stricture length (measured using barium enema), anastomotic stricture short diameter (the shortest distance of anastomotic stricture, measured stricture using barium enema), presence of stoma, intestinal epithelium preservation during ESt, and adverse events (AEs) (including perforation and bleeding) after ESt. Perforation was diagnosed based on patient symptoms, signs, and abdominal imaging. Bleeding included intra-procedure and post-procedure bleeding. Intra-procedure bleeding was defined as bleeding that required electrocoagulation or hemostatic clips to stop the bleeding during ESt. Post-procedure bleeding was defined as bleeding that required blood transfusion, embolization, or endoscopic or surgical intervention after ESt.

Procedure details and stricture data were obtained from patient medical records. Furthermore, post-procedure follow-up time was the time from index ESt to the last colonoscopy follow-up. Follow-up data were recorded as per routine clinical practice. Need for endoscopic or surgical reintervention was noted through procedure notes documented in the medical records, which were reviewed over the course of the study period.

### Endoscopic stricturotomy

**Fig. 1:**
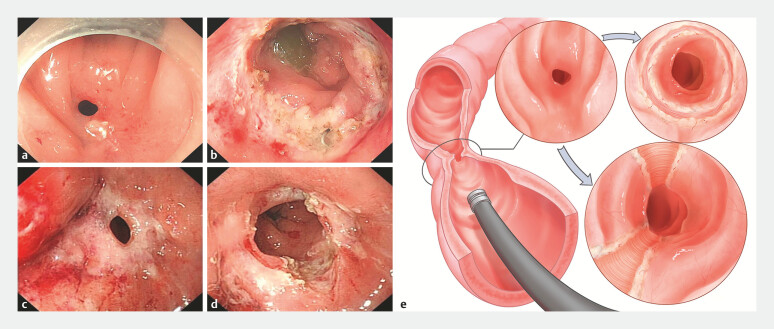
Endoscopic stricturotomy of anastomotic strictures. **a,b** Non epithelium-preserving incision. **c,d** Epithelium-preserving incision. **e** Illustration of the endoscopic stricturotomy methods.

ESt was performed with or without sedation and using a CF-H290 or PCF Q260J endoscope with a transparent cap (Olympus, Tokyo, Japan). The CF-H290 diameter is 12.2 mm, whereas that of the PCF Q260J is 10.5 mm (with a transparent cap attachment, the diameter is 12.4 mm). ESt was performed with an IT knife (KD611L, IT2; Olympus) or Dual knife (KD650Q; Olympus). ESt procedures were divided into epithelium-preserving and non-epithelium-preserving incisions, which were confirmed by two experienced endoscopists. An epithelium-preserving incision was defined as deep longitudinal incisions from the mucosa of the anal side across the anastomosis along the surface of the muscle. The epithelium between the incisions was preserved during the procedure. A non-epithelium-preserving incision was defined as a circumferential incision made in the stricture, and the scar tissue was excised in an arc from the incision along the lumen until adequate scope passage was achieved. Multiple radical incisions without epithelium preservation were considered non-epithelium-preserving incisions ( Fig. 1 ). Technical success was defined as ability to pass the endoscope through the stricture after index ESt.

Patients without stoma did not start oral feeding until 1 or 2 days after ESt. Antibiotics and hemostatic drugs were not routinely used prophylactically in patients after ESt. The next endoscopic assessment was planned 3 to 6 months after the first ESt. POBAS recurrence was defined as inability to pass an endoscope measuring approximately 12 mm in diameter through the stricture with or without stricture-related symptoms after the index ESt treatment was successful.

### Outcomes

The primary outcome was stricture recurrence. Secondary outcomes were technical success rate, postoperative AEs, and restricture-free rate (RFS).

### Statistical analysis

Statistical analysis was performed using R version 4.3.0. Quantitative variables conforming to the normal distribution were described using the mean ± standard deviation. Intergroup comparisons were made using Student *t* -tests. Quantitative variables not conforming to normal distribution were reported as the median, and the rank sum test was used for comparison between groups. Categorical variables were reported as numbers (percentages), and group differences were evaluated using the *χ* ^2^ test. Risk factors associated with POBAS recurrence were analyzed using univariate logistic regression analyses, and factors with *P* < 0.05 were included in multivariate logistic regression analysis to screen the independent risk factors. The value of the independent risk factors in the prognosis and early intervention of patients with anastomotic stricture was explored using RFS survival curve analysis.

## Results

**Fig. 2:**
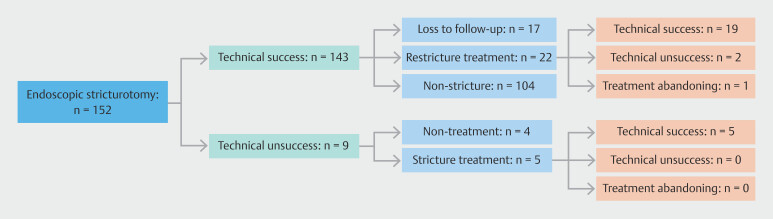
Flowchart of the study population.

A total of 152 patients met the study inclusion criteria. Fig. 2 depicts the study workflow, providing an overview of the study design.

### Demographic and clinical data

Mean patient age was 60.55 ± 12.74 years, and most patients were male (67.8%). Mean BMI was 22.08 ± 3.43 kg/m ^2^ . Up to 28.9% of the patients had anastomotic leakage. Of the patients, 71.7% had stoma and 37.5% received neoadjuvant therapy.

### Stricture characteristics

Median time from surgery to stricture was 6 months (range 1–71). Up to 18.4% of patients had anastomotic stricture length ≥ 1 cm. Mean anastomotic stricture short diameter was 0.59 ± 0.28 cm. Up to 59.8% of patients had anastomotic stricture distance from the anal margin ≤ 10 cm. Fifty-seven patients (37.5%) had a documented history of ESt with epithelium preservation.

### Outcomes

Immediate technical success was achieved in 91.4% of patients (143/152) ( Table 1 ). Postoperative AEs, including perforation and bleeding, were reported for 0.7% and 10.5% of the total number of patients, respectively. Of the 16 cases of intra-procedure bleeding, all were successfully managed with electrocoagulation or hemostatic clips, with no instances of post-procedure bleeding. One case of perforation resolved completely with conservative management, and no severe complications occurred. After excluding patients in whom index ESt was technically unsuccessful (n = 9) and who were lost to follow-up (n = 17), a total of 126 patients were included in the recurrent analysis with a 14.0-month follow-up (range 1.0–79.0). Twenty-two cases (17.5%) were diagnosed as recurrent stricture ( Table 2 ).

**Table:** **Table 1** Patient and anastomotic stricture characteristics.

Clinical characteristic	Overall (n = 152)
Age, years (mean ± SD)	60.55 ± 12.74
Sex	152 (100%)
Male	103 (67.8%)
Female	49 (32.2%)
BMI, kg/m ^2^ (mean ± SD)	22.08 ± 3.43
Time from surgery to stricture, months [median (range)]	6.00 (1.00–71.00)
Anastomotic stricture length, cm	152 (100%)
< 1	124 (81.6%)
≥ 1	28 (18.4%)
Anastomotic stricture short diameter, cm (mean ± SD)	0.59 ± 0.28
Anastomotic stricture distance from anal margin, cm	150 (98.7%)
> 10	59 (38.8%)
≤ 10	91 (59.9%)
Anastomotic leakage	152 (100%)
No	108 (71.1%)
Yes	44 (28.9%)
Enterostomy	147 (96.7%)
No	38 (25%)
Yes	109 (71.7%)
Neoadjuvant therapy	126 (82.9%)
No	94 (61.8%)
Yes	32 (21.1%)
Epithelium-preserving	152 (100%)
No	95 (62.5%)
Yes	57 (37.5%)
Adverse events	17 (11.2%)
Perforation	1 (0.7%)
Bleeding	16 (10.5%)
Technique success	152 (100%)
No	9 (8.6%)
Yes	143 (91.4%)
SD, standard deviation.	

**Table:** **Table 2** Comparison of clinical features between patients with recurrent and non-recurrent anastomotic stricture after ESt based on excluding patients with lost follow-up and unsuccessful technique.

Clinical characteristic	Overall (n = 126)	Recurrence (n = 22)	Non-recurrence (n = 104)	*P* value
Age, years (mean ± SD)	59.75 ± 12.67	63.00 ± 10.84	59.06 ± 12.97	0.144
Sex	126 (100%)	22 (100%)	104 (100%)	1.000
Male	84 (66.7%)	15 (68.2%)	69 (66.3%)	
Female	42 (33.3%)	7 (31.8%)	35 (33.7%)	
BMI, kg/m ^2^ (mean ± SD)	22.19 ± 3.50	23.09 ± 3.23	22.00 ± 3.54	0.169
Time from surgery to stricture, months [median (range)]	6.00 (1.00–46.00)	7.00 (3.00–32.00)	6.00 (1.00–46.00)	0.437
Anastomotic stricture length, cm	126 (100%)	22 (100%)	104 (100%)	< 0.05
< 1	106 (84.1%)	11 (50.0%)	95 (91.3%)	
≥ 1	20 (15.9%)	11 (50.0%)	9 (8.7%)	
Anastomotic stricture short diameter, cm (mean ± SD)	0.60 ± 0.27	0.45 ± 0.23	0.63 ± 0.27	< 0.05
Anastomotic stricture distance from anal margin, cm	124 (98.4%)	22 (100%)	102 (98.1%)	0.511
> 10	50 (39.7%)	7 (31.8%)	43 (41.3%)	
≤ 10	74 (58.7%)	15 (68.2%)	59 (56.7%)	
Anastomotic leakage	126 (100%)	22 (100%)	104 (100%)	0.984
No	89 (70.6%)	15 (68.2%)	74 (71.2%)	
Yes	37 (29.4%)	7 (31.8%)	30 (28.8%)	
Enterostomy	122 (96.8%)	21 (95.5%)	101 (97.1%)	1.000
No	34 (27.0%)	6 (27.3%)	28 (26.9%)	
Yes	88 (73.0%)	15 (68.2%)	73 (70.2%)	
Neoadjuvant therapy	107 (84.9%)	18 (81.8%)	89 (85.6%)	0.826
No	78 (61.9%)	14 (63.6%)	64 (61.5%)	
Yes	29 (23.0%)	4 (18.2%)	25 (24.0%)	
Epithelium-preserving	126 (100%)	22 (100%)	104 (100%)	< 0.05
No	78 (61.9%)	20 (90.9%)	58 (55.8%)	
Yes	48 (38.1%)	2 (9.1%)	46 (44.2%)	
Longitudinal incisions in ESt, [median (range)]	NA	3 (2.00–5.00)	NA	NA
Adverse events	15 (11.9%)	3 (13.6%)	12 (11.5%)	0.730
Perforation	1 (0.8%)	0 (0%)	1 (1.0%)	
Bleeding	14 (11.1%)	3 (13.6%)	11 (10.5%)	
ESt, endoscopic stricturotomy; NA, not available; SD, standard deviation.				

### Analysis of recurrence risk factors

The recurrence group had a significantly higher proportion of patients with anastomotic stricture length ≥ 1 cm (50.0%) than the non-recurrence group (8.7%) ( *P* < 0.05) ( Table 2 ). Patients with recurrence (0.45 ± 0.23 cm) also had a significantly smaller anastomotic stricture short diameter than patients without recurrence (0.63 ± 0.27 cm) ( *P* < 0.05). Furthermore, the recurrent group had a significantly smaller proportion of patients with intestinal epithelial preservation of ESt (9.1%) than the non-recurrent group (44.1%) ( *P* < 0.05).

### Comparison between epithelium-preserving and non-epithelium-preserving groups

Supplementary Table 2 compares clinical features between patients treated with epithelium-preserving ESt (n = 48) and non-epithelium-preserving ESt (n = 78). The epithelium-preserving group had a significantly lower proportion of strictures ≥1 cm (6.2% vs. 21.8%, *P* < 0.05) and a larger mean stricture short diameter (0.78 ± 0.20 cm vs. 0.48 ± 0.24 cm, P < 0.05). No significant differences were observed in age, sex, BMI, time from surgery to stricture, anastomotic leakage, stoma presence, or neoadjuvant therapy between the two groups. AEs were comparable (8.3% vs. 14.1%, *P* = 1.000).

### Factors associated with recurrence after ESt

Univariate logistic regression analysis results demonstrated that stricture length, stricture short diameter, and whether the intestinal epithelium was preserved during ESt were closely related to stricture recurrence after ESt ( *P* < 0.05). Multivariate logistic regression analysis results demonstrated that anastomotic stricture length ≥ 1 cm and non-epithelium-preservation during ESt were independent risk factors for recurrent anastomotic stricture after ESt ( *P* < 0.05) ( Table 3 ).

**Table:** **Table 3** Risk factors for restricture following ESt.

Clinical characteristic	Univariate analysis			Multivariate analysis		
	Hazard Ratio (HR)	95% confidence interval (CI)	*P* value	Hazard Ratio (HR)	95% confidence interval (CI)	*P* value
Age, years	4.137	0.783–21.851	0.0945	–	–	–
Sex	1.376	0.560–3.378	0.486	–	–	–
BMI, kg/m ^2^	3.227	0.499–20.860	0.219	–	–	–
Time from surgery to stricture, months	1.335	0.836–2.133	0.226	–	–	–
Anastomotic stricture length, cm	7.009	3.034–16.192	5.182e-06	5.2399	2.030–13.526	< 0.05
Anastomotic stricture short diameter, cm	0.0952	0.0199–0.455	0.00322	0.880	0.120–6.453	0.900
Anastomotic stricture distance from anal margin, cm	0.600	0.244–1.472	0.265	–	–	–
Anastomotic leakage	1.119	0.456–2.745	0.806	–	–	–
Enterostomy	0.960	0.372–2.476	0.933	–	–	
Neoadjuvant therapy	0.756	0.249–2.297	0.622	–	–	–
Epithelium-preserving	0.144	0.0336–0.616	0.00897	0.205	0.0434–0.969	< 0.05
Adverse events	2.286	0.675–7.740	0.184	–	–	–
BMI, body mass index; ESt, endoscopic stricturotomy.						

**Fig. 3:**
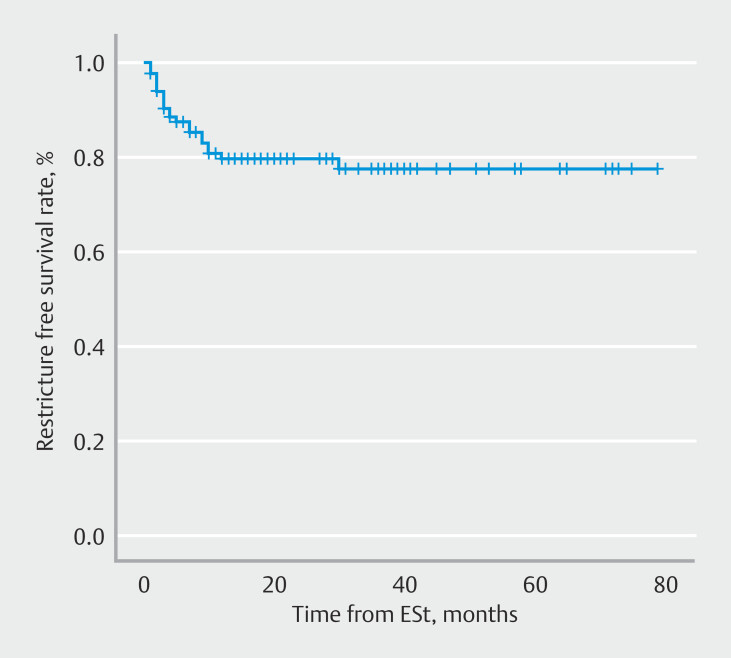
Kaplan-Meier analysis of RFS in 126 patients with follow-up data.

**Fig. 4:**
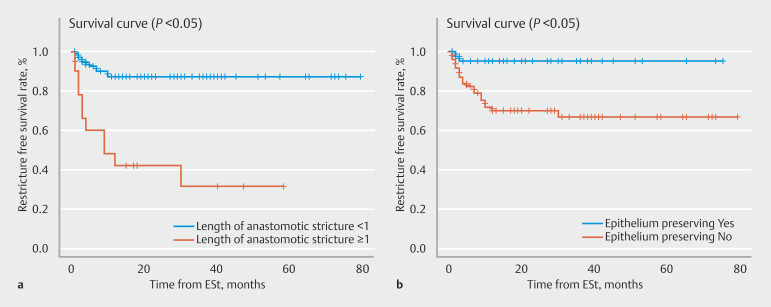
Kaplan Meier analysis of RFS in patients according to stricture length ( **a** ) and epithelium preservation status ( **b** ) .

A total of 126 patients with ESt for initial anastomotic stricture had follow-up data. The interval between the index ESt and the first recurrence was calculated to construct the survival curves ( Fig. 3 ). RFS was evaluated using Kaplan-Meier analysis. The cumulative RFS rate was 82.53% (Supplementary Table 1). Survival analysis between the groups revealed that patients with stricture length ≥ 1 cm had significantly worse RFS ( Fig. 4 **a** ) ( *P* < 0.05). Furthermore, patients with non-epithelium preservation at ESt had significantly worse RFS ( Fig. 4 **b** ) ( *P* < 0.05).

## Discussion

In this retrospective study, we evaluated 152 patients who were treated with ESt for first-time POBAS that occurred after surgery for CRC. Immediate technical success was achieved in 91.4% of patients, with few AEs. The cumulative RFS rate was 82.53%. Anastomotic stricture length ≥ 1 cm and non-epithelium-preservation during ESt were independent risk factors for POBAS recurrence after index ESt.

Anastomotic stricture is a prevalent complication following radical CRC surgery. Although POBAS is not always symptomatic, frequently reported symptoms include pain, cramps, constipation, fractionated evacuation, and abdominal distension, which greatly affect patient quality of life 3 9 . Interventional endoscopy has expanded to become effective management for POBAS, whereas some patients with POBAS may be cured through common treatment methods, such as EBD. For example, although EBD demonstrates a high initial success rate, patients often require repeat dilations (up to 88%), with rare cases of serious complications after EBD 10 . ESt was first applied to treat primary and secondary inflammatory bowel disease (IBD)-related stricture effectively and safely. ESt has been increasingly used for treating POBAS in non-IBD in recent years. Zhang et al. evaluated the outcome of ESt in management of IBD and non-IBD associated strictures 11 . Non-IBD patients appeared to have a greater need for subsequent surgery but a lower complication rate than IBD patients. In the present study, immediate technical success was recorded in 91.4% of patients, and the cumulative RFS was 82.53%. Therefore, ESt demonstrated promising results for treating POBAS of CRC.

Although ESt is effective in treating POBAS of CRC, we determined that 17.5% of our patients experienced restricture after initially successful ESt. We wanted to clarify risk factors for recurrent stricture after ESt. In IBD patients, ESt was indicated for short (≤ 4 cm), predominantly fibrotic/mixed strictures as a primary therapy or as salvage therapy in refractory strictures 12 . ESt is preferable in non-angulated strictures, where the cut depth can be controlled with a stable endoscope tip 7 . Few studies have focused on risk factors for recurrent stricture after ESt. Kim et al. reported that patients receiving neoadjuvant radiotherapy were the most prone to restricture after an EBD 10 . In the present study, neoadjuvant therapy was not a risk factor for restricture after ESt. We determined that anastomotic stricture length ≥ 1 cm was an independent risk factor for POBAS recurrence after index ESt. This result was consistent with a previous study at our center. Therefore, stricture length must be assessed accurately when considering ESt in POBAS of CRC.

The main advantages of ESt over EBD are endoscopist full control of electroincision location and depth and availability of various cutting approaches, such as radial, horizontal, and circumferential incisions. However, no standard ESt procedure has been established. Because scar tissue is excised in an arc from the incision along the lumen during ESt, ESt is considered a viable alternative to EBD, featuring superior long-term effectiveness and reduced reintervention rates 13 14 15 . However, the present study determined that a non-epithelium-preserving incision was independently associated with POBAS recurrence after ESt of CRC. Therefore, in addition to factors from the patient’s anastomosis, we should also focus on the effect of the incision method on anastomotic stricture recurrence. Similar phenomena and mechanisms have been studied in procedure for prolapse and hemorrhoids (PPH) treatment of hemorrhoids. PPH could lead to anorectal stricture, which might be caused by the entire circular anastomosis nail 16 . In the tissue selection technique (TST), a specially designed anoscope is used to remove only the rectal mucosa protruding above the hemorrhoids to protect the normal mucosa between the resected mucosa, overcoming the limitations and weaknesses of PPH, and has a much lower stricture rate than PPH 17 . Gu et al. developed complete anal canal epithelial preservation surgery, which protects the anal canal epithelium by making curved incisions on external hemorrhoids 18 .

Similar to anorectal stricture mechanisms, anastomotic stricture formation may be associated with inflammatory hyperplasia of the scar. Therefore, we included epithelium preservation during ESt in the risk factor analysis of POBAS recurrence. Patients whose epithelium was preserved during ESt had a higher RFS rate, indicating that improving stricture treatment might reduce risk of POBAS recurrence, and has certain clinical application prospects. Endoscopists have also attempted to improve the incision technique to achieve better results. Ebigbo et al. successfully performed peroral submucosal endoscopic stricturotomy, which is a novel third-space approach preserving the whole epithelium of anastomosis, for a complex anastomotic stricture 19 .

One limitation of this study is its design, because it was single-center and retrospective. In addition, although we presented a large sample size report on ESt for POBAS after surgery for CRC, the follow-up time of patients after ESt was relatively short. Third, detailed information about neoadjuvant therapies (e.g., chemotherapy, radiotherapy), adjuvant therapies, surgical techniques (e.g., type of anastomosis), and operative approaches was incompletely documented in some patient records, limiting our ability to assess their potential influence on stricture recurrence. However, from a novel perspective (based on modified ESt to prevent POBAS recurrence), we analyzed independent risk factors for stricture recurrence of POBAS of CRC, and provided reliable data support for adjusting endoscopic protocols for stricture treatment and improving patient postoperative quality of life.

## Conclusions

ESt is a safe and effective technique to treat patients with POBAS after surgery for CRC. In addition to anastomotic stricture length ≥ 1 cm, non-epithelium-preserving incision during ESt is a newly discovered independent risk factor for stricture recurrence. Further studies of the standard procedure of ESt in treating patients with POBAS are needed.

## Supplementary Material

Supplementary Material Zusatzmaterial
