# Supplementary material for: Epithelium-preserving stricturotomy is effective for improving postoperative benign anastomotic stricture associated with colorectal surgery
Source: Endosc Int Open. 2025 May 16;13:a25923133. doi: 10.1055/a-2592-3133 (PMC12090975; doi:10.1055/a-2592-3133)
Supplement: Supplementary file 1 — Supplementary Material [file 10-1055-a-2592-3133_25937806.pdf]

Supplementary Table 1 Proportion of current cumulative restricture-free analysis.

Patient ID	Time	Status	Proportion of current cumulative restricture-free analysis	Number of cumulative cases	Number of remaining cases
1	1	1	0.992063492	1	125
2	1	1	0.984126984	2	124
3	1	1	0.976190476	3	123
4	1	0	0.976190476	3	123
5	1	0	0.976190476	3	123
6	1	0	0.976190476	3	123
7	1	0	0.976190476	3	123
8	1	0	0.976190476	3	123
9	1	0	0.976190476	3	123
10	1	0	0.976190476	3	123
11	1	0	0.976190476	3	123
12	1	0	0.976190476	3	123
13	1	0	0.976190476	3	123
14	1	0	0.976190476	3	123
15	1	0	0.976190476	3	123
16	1	0	0.976190476	3	123
17	1	0	0.976190476	3	123
18	2	1	0.968253968	4	122
19	2	1	0.96031746	5	121
20	2	1	0.952380952	6	120
21	2	1	0.944444444	7	119
22	2	0	0.944444444	7	119
23	2	0	0.944444444	7	119
24	2	0	0.944444444	7	119

Endosc Int Open | DOI 10.1055/a-2592-3133 | © The Author(s).

25	2	0	0.944444444	7	119
26	3	1	0.936507937	8	118
27	3	1	0.928571429	9	117
28	3	1	0.920634921	10	116
29	3	1	0.912698413	11	115
30	3	0	0.912698413	11	115
31	3	0	0.912698413	11	115
32	4	1	0.904761905	12	114
33	4	1	0.896825397	13	113
34	4	0	0.896825397	13	113
35	4	0	0.896825397	13	113
36	4	0	0.896825397	13	113
37	4	0	0.896825397	13	113
38	5	1	0.888888889	14	112
39	5	0	0.888888889	14	112
40	5	0	0.888888889	14	112
41	5	0	0.888888889	14	112
42	6	0	0.888888889	14	112
43	6	0	0.888888889	14	112
44	6	0	0.888888889	14	112
45	7	1	0.880952381	15	111
46	7	1	0.873015873	16	110
47	7	0	0.873015873	16	110
48	7	0	0.873015873	16	110
49	8	0	0.873015873	16	110
50	8	0	0.873015873	16	110
51	9	1	0.865079365	17	109
52	9	1	0.857142857	18	108
53	10	1	0.849206349	19	107

54	10	1	0.841269841	20	106
55	10	0	0.841269841	20	106
56	10	0	0.841269841	20	106
57	11	0	0.841269841	20	106
58	12	1	0.833333333	21	105
59	12	0	0.833333333	21	105
60	12	0	0.833333333	21	105
61	12	0	0.833333333	21	105
62	13	0	0.833333333	21	105
63	14	0	0.833333333	21	105
64	14	0	0.833333333	21	105
65	15	0	0.833333333	21	105
66	15	0	0.833333333	21	105
67	15	0	0.833333333	21	105
68	15	0	0.833333333	21	105
69	15	0	0.833333333	21	105
70	16	0	0.833333333	21	105
71	17	0	0.833333333	21	105
72	18	0	0.833333333	21	105
73	18	0	0.833333333	21	105
74	18	0	0.833333333	21	105
75	19	0	0.833333333	21	105
76	19	0	0.833333333	21	105
77	20	0	0.833333333	21	105
78	20	0	0.833333333	21	105
79	21	0	0.833333333	21	105
80	21	0	0.833333333	21	105
81	22	0	0.833333333	21	105
82	22	0	0.833333333	21	105

83	23	0	0.833333333	21	105
84	27	0	0.833333333	21	105
85	27	0	0.833333333	21	105
86	28	0	0.833333333	21	105
87	28	0	0.833333333	21	105
88	29	0	0.833333333	21	105
89	30	1	0.825396825	22	104
90	30	0	0.825396825	22	104
91	30	0	0.825396825	22	104
92	31	0	0.825396825	22	104
93	31	0	0.825396825	22	104
94	33	0	0.825396825	22	104
95	33	0	0.825396825	22	104
96	35	0	0.825396825	22	104
97	35	0	0.825396825	22	104
98	36	0	0.825396825	22	104
99	36	0	0.825396825	22	104
100	37	0	0.825396825	22	104
101	38	0	0.825396825	22	104
102	38	0	0.825396825	22	104
103	39	0	0.825396825	22	104
104	39	0	0.825396825	22	104
105	39	0	0.825396825	22	104
106	40	0	0.825396825	22	104
107	40	0	0.825396825	22	104
108	41	0	0.825396825	22	104
109	42	0	0.825396825	22	104
110	42	0	0.825396825	22	104
111	45	0	0.825396825	22	104

112	47	0	0.825396825	22	104
113	51	0	0.825396825	22	104
114	51	0	0.825396825	22	104
115	53	0	0.825396825	22	104
116	57	0	0.825396825	22	104
117	58	0	0.825396825	22	104
118	64	0	0.825396825	22	104
119	65	0	0.825396825	22	104
120	65	0	0.825396825	22	104
121	71	0	0.825396825	22	104
122	72	0	0.825396825	22	104
123	73	0	0.825396825	22	104
124	73	0	0.825396825	22	104
125	75	0	0.825396825	22	104
126	79	0	0.825396825	22	104

Supplementary Table 2 Comparison of clinical features between patients with epithelium-preserving and nonepithelium-preserving in ESt based on excluding patients lost to follow-up and in whom ESt was unsuccessful.

**Clinical characteristics**	**Overall (n = 126)**	**Epithelium-preserving (n = 48)**	**Non-epithelium-preserving (n = 78)**	***P* value**
Age, years (mean ± SD)	59.75 ± 12.67	60.98 ± 12.33	58.99 ± 12.89	0.389
Sex	126 (100%)	48 (100%)	78 (100%)	0.846
Male	84 (66.7%)	33 (68.8%)	51 (65.4%)	
Female	42 (33.3%)	15 (31.3%)	27 (34.6%)	
BMI, kg/m^2 (mean ± SD)	22.19 ± 3.50	21.85 ± 2.91	22.40 ± 3.82	0.36
Time from surgery to stricture, months [median (range)]	6.00 (1.00 - 46.00)	6.50 (1.00 - 46.00)	6.00 (1.00 - 32.00)	0.732
Anastomotic stricture length, cm	126 (100%)	48 (100%)	78 (100%)	< 0.05
< 1	106 (84.1%)	45 (93.8%)	61 (78.2%)	
≥ 1	20 (15.9%)	3 (6.2%)	17 (21.8%)	
Anastomotic stricture short diameter, cm (mean ± SD)	0.60 ± 0.27	0.78 ± 0.20	0.48 ± 0.24	< 0.05
Anastomotic stricture distance from anal margin, cm	124 (98.4%)	47 (97.9%)	77 (98.7%)	0.83

>10	50 (39.7%)	21 (43.8%)	48 (61.5%)	
≤10	74 (58.7%)	26 (54.1%)	29 (37.2%)	
Anastomotic leakage	126 (100%)	48 (100%)	78 (100%)	0.811
No	89 (70.6%)	35 (72.9%)	54 (69.2%)	
Yes	37 (29.4%)	13 (27.1%)	24 (30.8%)	
Enterostomy	122 (96.8%)	46 (95.8%)	76 (97.4%)	0.894
No	34 (27.0%)	12 (25.0%)	22 (28.2%)	
Yes	88 (69.8%)	34 (70.8%)	54 (69.2%)	
Neoadjuvant therapy	107 (84.9%)	42 (87.5%)	65 (83.3%)	0.959
No	78 (61.9%)	30 (62.5%)	48 (61.5%)	
Yes	29 (23.0%)	12 (25.0%)	17 (21.8%)	
Adverse events	15 (11.9%)	4 (8.3%)	11 (14.1%)	1
Perforation	1 (0.8%)	0 (0%)	1 (1.3%)	
Bleeding	14 (11.1%)	4 (8.3%)	10 (12.8%)	

ESt, endoscopic stricturotomy; SD, standard deviation.

Thieme

Supplementary Figure 1

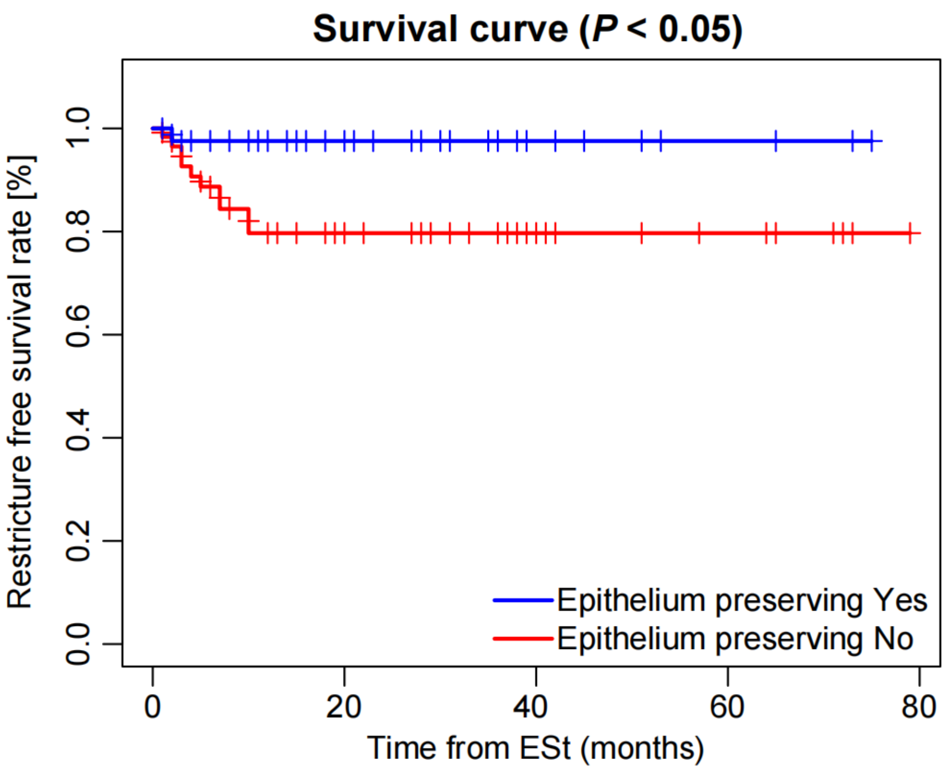

Endosc Int Open | DOI 10.1055/a-2592-3133 | © The Author(s).
